# Unveiling the complexity of civil service effectiveness index: An asymmetric and ANN modeling

**DOI:** 10.1016/j.heliyon.2024.e39776

**Published:** 2024-10-24

**Authors:** Munshi Muhammad Abdul Kader Jilani, Md Mominur Rahman, Md Abdul Latif, Nasim Ahmed

**Affiliations:** a Department of Human Resource Management, Bangladesh Institute of Governance and Management (BIGM), University of Dhaka (Affiliated), Bangladesh; b Bangladesh Institute of Governance and Management (BIGM), University of Dhaka (Affiliated), Bangladesh; c Training and Development Wing, Bangladesh Institute of Governance and Management (BIGM), University of Dhaka (Affiliated), Bangladesh; d Department of Public Policy and Governance, Bangladesh Institute of Governance and Management (BIGM), University of Dhaka (Affiliated), Bangladesh

**Keywords:** Civil service effectiveness, Core executive functions, Mission support facilities, Service delivery functions, Attributes, Hybrid analytical framework

## Abstract

Unraveling the factors influencing civil service effectiveness becomes imperative in an era marked by escalating demands for efficient governance. This study attempts to meet this necessity by delving into the complex dynamics among core executive functions, mission support facilities, service delivery functions, and attributes, aiming to elucidate their collective impact on civil service effectiveness. Utilizing a unique methodological blend of Fuzzy-set Qualitative Comparative Analysis (fsQCA) and Artificial Neural Networks (ANN), it delves into the relationships among core executive functions, mission support facilities, service delivery functions, and attributes within the International Civil Service Effectiveness (InCiSE) Index framework. The research uses a configurational model that optimizes CSE and assesses the relative importance of various components. The study reveals significant correlations among the variables. It indicates that all CSE indicators influence but are not equally important in triggering the effectiveness of civil service administration. Key configurations, such as integrating strategic governance with mission support functions and high-level strategy with operational execution, are critical for enhancing civil service effectiveness. It underscores the importance of prioritizing core executive functions and attributes to improve civil service administration. Theoretically, the study enriches contingency theory and contributes to the civil service and administration literature by integrating a configurational approach with machine learning insights. Practically, it provides actionable insights for governance improvement, promoting the application of innovative methodologies in public service to enhance organizational environment and civil service capacities. Original in its approach, this study fills a gap in the literature by applying a hybrid fsQCA-ANN model to explore the configurational and construct ranks influencing civil service effectiveness, offering an inclusive analysis that triangulates qualitative and quantitative data. The findings indicate that civil service effectiveness is highly complex because of its process, involvement of diverse backgrounds of civil servants, critical understanding about the key pillars of good governance and service delivery mechanism. Thus, the findings advance academic understanding and provide practical strategies for policymakers and practitioners to foster better governance through targeted interventions that enhance transparency, accountability, and responsiveness in civil service organizations.

## 1 Introduction

In an era marked by globalization, rapid technological advancements, and complex socio-economic challenges, the imperative for effectiveness in civil service has become paramount [[1], [2], [3], [4]]. Civil service acts as the backbone of governance structures worldwide, playing a pivotal role in policy formulation, service delivery, and societal development [5,6]. Their role is characterized by a commitment to professionalism and adaptability, which are essential for navigating the complexities of modern governance. However, amid growing demands for accountability, transparency, and responsiveness, civil service encounters numerous challenges in meeting the evolving needs of both citizens and governments [7,8]. These challenges are not only limited to navigating political dynamics and bureaucratic hurdles [9] but also addressing resource constraints [10], adapting to technological disruptions [11], ensuring inclusivity and diversity [12], and upholding integrity and ethical standards [1]. As such, understanding the complexities and intricacies of civil service effectiveness is crucial for devising strategies to overcome these challenges and fostering governance systems that are efficient, equitable, and responsive to the needs of the state.

The measurement of civil service effectiveness (CSE) has long been a critical focus in public administration, driven by the need to improve public sector performance and accountability. Initial efforts in the mid-20th century, such as those by Simon and Barnard [13] and Waldo [14], emphasized the principles of effective governance but were largely theoretical, and lacked of empirical tools for assessing civil service performance. The emergence of ‘New Public Management’ in the 1980s and 1990s, highlighted by Hood [15], marked a shift toward quantitative approaches, introducing specific metrics for evaluating efficiency and accountability. In the early 2000s, the development of composite indices gained momentum, with the World Bank's ‘Worldwide Governance Indicators’ Kaufmann, Kraay [16] pioneering efforts to measure government effectiveness, setting the stage for more focused studies on civil services. Subsequent research, particularly by Kohtamäki and Olsson [17], advocated for a multidimensional framework that integrated input, output, and outcome measures, further refined the methodologies used in constructing the Civil Service Effectiveness Index (CSEI). Recently, methods for measuring the CSEI have advanced significantly, incorporating sophisticated statistical techniques like factor analysis and structural equation modeling, as highlighted by Peters and Peters [18] and Lodge and Wegrich [19]. These innovations have led to more precise measurements and deeper insights into civil service effectiveness. So, integrating big data and real-time analytics, as explored by Mittal [20], marks a shift from traditional, static methods to more dynamic and responsive approaches. This evolution reflects the broader trend in public administration towards empirical, data-driven research, providing policymakers with more robust tools to enhance governance and public sector outcomes.

The International Civil Service Effectiveness (InCiSE) Index, a collaborative effort between the Blavatnik School of Government, the Institute for Government, and supported by the UK Civil Service and funded by the Open Society Foundations, addresses the critical need for a comprehensive assessment of civil service effectiveness globally [1]. By evaluating core functions, i.e., policymaking, fiscal management, and service delivery, alongside attributes like integrity, openness, and innovation, InCiSE provides a nuanced understanding of civil service performance [1]. Indeed, understanding how the core executive functions, mission support facilities, service delivery functions, and attributes designed by InCiSE impact civil service effectiveness is crucial for enhancing governance outcomes. Each component plays a vital role in shaping the overall performance of civil services. For instance, core executive functions like policymaking and crisis management directly influence the government's ability to respond to societal needs and challenges. Similarly, mission support facilities such as procurement and HR management are essential for ensuring the efficient operation of civil service organizations [1]. In addition, service delivery functions like tax administration and digital services directly influence citizens' experiences with government services. Additionally, attributes such as integrity, openness, and staff engagement contribute significantly to civil services' organizational culture and effectiveness [1]. Therefore, examining how these functions and attributes interact and affect CSE is essential for identifying areas of improvement, enhancing accountability, and fostering better governance.

The objectives of the study are to identify the causal relationship between core executive functions, to discover the impact of mission support facilities, to test the relationship between service delivery functions, to determine the effect of attributes, and to uncover necessary and sufficient conditions and configurations for achieving high levels of civil service effectiveness, providing valuable insights for policymakers and practitioners to enhance governance outcomes. The study offers significant contributions to civil service effectiveness, factors driving performance of civil service, improving governance; contingency theory, providing a nuanced understanding of organizational dynamics; and to the methodological combination of fuzzy-set Qualitative Comparative Analysis (fsQCA) and Artificial Neural Network (ANN) modeling. Given the above-presented logic, the scholarly focus on CSE has become a significant area of research over the past few decades. The following research questions (RQ) should be answered to address this issue comprehensively.

RQ1: How do core executive functions (CEF), mission support facilities (MSF), service delivery functions (SDF), and their attributes impact civil service effectiveness?
RQ2: How does fsQCA uncover causal pathways to civil service effectiveness?
RQ3: In what ways does ANN modeling deepen the understanding of factors influencing civil service effectiveness?
RQ4: When analyzing civil service effectiveness, what are the fundamental topologies and implications derived from fsQCA and ANN techniques?

## 2 Theoretical framework

The study of civil service effectiveness has relied on various theoretical frameworks. Research indicates that a theoretical approach across different contexts can enhance explanatory power by creating an integrated model [21]. Despite these theories' diverse assumptions and functional attitudes, empirical evidence shows that combining them provides comprehensive insights into civil service administration. As a result, Fiedler [22] developed a contingency theory specifically to explain civil service effectiveness. Contingency theory proposes that organizational effectiveness is contingent upon the fit between the organization's structure, processes, and the external environment [23,24]. It suggests that there is no one-size-fits-all approach to organizational design or management; instead, organizations must adapt their structures and strategies to align with the specific demands of their context [23]. Contingency theory emphasizes that various situations require varied responses, and the effectiveness of organizational practices depends on how well they match the demands of the environment [24,25]. In the context of this study, contingency theory provides a valuable lens through which this study attempts to interpret the relationships between core executive functions, mission support facilities, service delivery functions, attributes, and civil service effectiveness. The theory suggests that the efficacy of civil services may vary depending on the alignment between these components and the broader institutional and societal context in which they operate [25]. For example, the impact of core executive functions like policymaking and crisis management on civil service effectiveness may be contingent upon political stability, economic conditions, and institutional capacity [26]. Similarly, the effectiveness of mission support facilities such as procurement and HR management may depend on organizational structures, resource allocation, and technological capabilities.

Furthermore, contingency theory suggests that attributes such as integrity, openness, and staff engagement may influence the relationship between functions and CSE. For instance, a high level of integrity and openness within the civil service may enhance the effectiveness of core functions by fostering transparency, accountability, and public trust [27]. Similarly, strong staff engagement and capabilities may enable civil service organizations to adapt more effectively to changing demands and deliver services more efficiently [26]. By considering these contingencies, the study can provide insights into the conditions under which different combinations of functions and attributes contribute to high levels of civil service effectiveness, eventually informing strategies for improving governance outcomes.

## 3 Literature review, relationship and its proposition

### 3.1 Relationship between core executive functions and civil service effectiveness

An effective and efficient civil service is the cornerstone of a nation's advancement and prosperity. As the InCiSE report outlines, the core executive functions encapsulate the synergistic relationship between policymaking, fiscal and financial management, regulation, and crisis and risk management [28]. This study underscores the pivotal role of effective policymaking in determining civil service effectiveness by establishing governmental endeavours' trajectory and focal points [29]. Civil service displays adeptness in policy analysis, formulation, and execution and tends to realize desired outcomes more readily. Studies by Dorren and Wolf [30] indicate that civil service that embrace evidence-based policymaking and foster stakeholder engagement demonstrates higher effectiveness. In addition, the capacity to evolve and adjust policies by shifting circumstances emerges as a critical determinant of civil service efficacy [31]. With this view, research indicates that civil service equipped with budgetary frameworks, efficient revenue collection mechanisms, and prudent expenditure oversight ascends to higher levels of effectiveness [32].

Moreover, transparency and accountability in financial management contribute significantly to CSE, as they enhance public trust and confidence [33]. Further, the research of Elsawy and Al-Ghurabli [34] highlights the indispensable role of fiscal sustainability and visionary planning in strengthening the effectiveness of civil service operations. Previous studies in this field reveal that clear, consistent, and predictable regulations significantly enhance CSE when adequately designed and effectively implemented [35]. These services prioritizing regulatory rationalization, streamlining, and harmonization are generally effective in improving business competitiveness and fostering innovation [36]. In the broader framework of governance, efficient and well-designed regulatory impact assessment and monitoring mechanisms are crucial for evaluating the effectiveness of regulations and identifying areas for improvement.

Civil services must adeptly anticipate, prepare for, respond to, and recover from crises and risks spanning natural disasters, pandemics, and security threats [4]. Crisis and risk management research in civil service underscores the crucial role of proactive risk assessment, agile contingency planning, and seamless coordination [37]. These pillars strengthen CSE and steer it through challenging circumstances, while services that demonstrate agility, resilience, and interagency coordination excel in crisis mitigation. Other research, such as those by Cedergren and Hassel [38] and Haase [39], point out leadership, communication, and public engagement as vital in fostering trust and confidence in crisis management efforts. Research consistently demonstrates that the ability of civil services to perform CEF is essential for fostering good governance and improving civil service effectiveness for public welfare.

Proposition 1 *Enhancing civil service effectiveness requires optimizing CEF in areas such as policymaking*, *fiscal and financial management*, *regulation*, *and crisis and risk management*.

### 3.2 Impact of mission support facilities on CSE

MSFs are vital resources that equip organizations with the necessary tools to achieve objectives and address challenges, including compliance and promoting social and economic progress [28]. Their crucial function, like HR management and procurement, enhances civil service efficacy and productivity, emphasize their importance in research. The efficacy of the civil service is contingent upon implementing effective human resource management practices, which serve as a fundamental element in maximizing the capabilities of personnel [40]. Woo and Kim [41] coin that civil service that possesses healthy HR strategies, including effective recruitment, thorough training, precise performance evaluation, and career development, tends to achieve higher levels of effectiveness. Further analysis reveals the significant impact of investing in civil service training and development initiatives [42], which not only enhances skill sets but also fosters increased job satisfaction, thereby creating a workforce positioned for greater productivity and effectiveness [43]. Additionally, many scholarly studies underline the critical importance of HR policies promoting diversity, equity, and inclusion [44]. It shapes the frame of the civil service, rendering it more effective and inherently more attuned and responsive to the multifaceted needs of diverse stakeholders.

Furthermore, Basheka [45] coins efficient procurement processes that play a pivotal role in facilitating the civil service acquisition of essential resources and services, thereby underpinning the successful execution of their mandates. Research findings prove that streamlined procurement processes, including transparent bidding procedures, vendor selection criteria, and contract management, contribute to cost savings and improved service delivery within the civil service [45]. Moreover, academic researchers delve into the need to maintain procurement integrity and accountability as crucial in reducing corruption and promoting public confidence in government institutions [46]. From this perspective, civil service prioritizes ethical procurement practices and adopts innovative approaches, i.e., e-procurement, which are better placed to achieve value for money and enhance overall effectiveness. In essence, the research conducted by Kyrychok, Harbuza [47] alongside Althabatah, Yaqot [48] scores the pivotal role of MSF, such as HR management and procurement, in shaping civil service effectiveness. By strategically capitalizing on these areas and implementing best practices, civil service organizations can raise performance standards, strengthen accountability mechanisms, and ultimately achieve better outcomes for their citizens. Based on the review, we propose the following proposition as we begin our journey.

Proposition 2 *Strengthening the capacity of the civil service effectiveness depends on substantial MSF such as human resources management and procurement*.

### 3.3 Role of service delivery functions in enhancing CSE

According to Bassey, Mulligan [49], the integration of efficient tax administration and digital services improves service performance by fostering revenue creation, ensuring economic stability, promoting compliance, building public trust, increasing accessibility, enhancing efficiency, encouraging citizen engagement, driving innovation, and enabling the effective utilization of data. These functions are essential in strengthening government performance and addressing the requirements of citizens in a progressively digital and interconnected world. Empirical studies of Lan [50] highlight the critical function of effective tax administration, promoting revenue mobilization and nurturing economic growth. Civil service that implements efficient tax-collecting procedures, effective enforcement strategies, and proactive efforts to educate taxpayers are more likely to achieve increased effectiveness [51]. Further, studies by Saragih, Reyhani [52] support the modernization of tax systems, utilizing technological advancements to improve compliance and alleviate administrative burdens to support the efficacy of tax administration. Moreover, published literature and academic discussions indicate that transparency, fairness, and accountability in tax administration are underscored, as they are fundamental pillars that promote public trust and adherence [53].

Moreover, the advent of digital government services holds promise for substantial enhancements in civil service effectiveness, chiefly through improved accessibility, efficiency, and responsiveness Idzi and Gomes [54]. Consequently, the scholarly review by Patergiannaki and Pollalis [55] and Clarke [56] indicates that civil services embracing digital transformation endeavors, such as service delivery platforms, e-government portals, and digital identity systems, experience improvements in service quality and citizen satisfaction. There are currently few thorough, scholarly studies available on digital technologies and services in the context of public management research. Notably, studies [57,58] emphasize the importance of user-centric design principles, interoperability standards, and data privacy and security measures ensuring the success of digital service initiatives, as cited by Vashkevich, Barykin [59]. Furthermore, scholarly discussions state the pivotal roles of capacity-building initiatives, stakeholder engagement strategies, and regulatory frameworks in facilitating the seamless adoption and proficient utilization of digital services within civil service organizations [50]. So, civil service organizations can improve their capacity to provide citizens with high-quality services and effectively address their ever-changing needs by strategically implementing best practices, utilizing the transformative power of technology, and cultivating an environment of openness and accountability. Based on the perspective mentioned earlier, the following proposition has been formulated.

Proposition 3 *The success of civil service effectiveness relies on the strength of SDF*, *as demonstrated by efficient tax administration and digital services*.

### 3.4 Influence of attributes on strengthening civil service effectiveness

The foundation of successful and responsive governance comprises qualities like integrity, openness, capabilities, and inclusivity. These qualities are closely associated with the effectiveness of the civil service [28]. Integrity ensures that civil servants uphold ethical standards, fostering trust among citizens and stakeholders while reducing corruption and enhancing accountability [60]. Taamneh, Yakoub [61] mention that civil service demonstrates integrity with greater responsibility, openness, and moral conduct in their day-to-day activities. As a result, integrity is linked to reduction of corruption and improvement of governance outcomes, contribution to overall organizational effectiveness and building public trust in government institutions. Openness enhances transparency, enabling citizen engagement and participation in decision-making, ultimately improving service delivery outcomes [21]. Well-studied topics in the literature reveal that civil services that prioritize transparency are more capable of meeting the demands of the public, encouraging responsibility, and augmenting public involvement in choices [33]. Hence, openness fosters trust between government and citizens, ultimately improving service delivery and governance outcomes. Capabilities empower civil service personnel with the skills and competencies necessary to fulfil their roles effectively, driving innovation, adaptability, and organizational effectiveness [62]. Supporting evidence from the literature review [47,63] exposes that the implementation of professional development programs, education, and training initiatives tailored to enhancing the capabilities of the civil service contributes to improved organizational effectiveness and performance. So, civil services with a diverse and skilled workforce are better equipped to tackle complex challenges, innovate, and adapt to changing circumstances, improving overall effectiveness.

Recent literature of Bidstrup, Gabova [64] suggests that inclusiveness invites many voices and enriches policymaking and service delivery, resulting in solutions that resonate with all citizens and foster a more profound sense of satisfaction and trust in government institutions, enhancing a stronger bond between the civil service and the communities it serves. Findings of previous studies also posit that civil service that encourages inclusivity is more adept at addressing the requirements of various communities, resulting in improved service delivery outcomes and increased citizen contentment [21]. To sum up, inclusiveness fosters a culture of belonging and participation within civil service organizations, which in turn enhances employee morale, productivity, and effectiveness. Together, these attributes contribute to civil service effectiveness, which is effective in achieving its objectives and accountable, transparent, and inclusive in its operations [65].

Proposition 4 *Enhancing civil service effectiveness depends on attributes i*.*e*., *integrity*, *openness*, *capabilities*, *and inclusivity*.

**Fig. 1:**
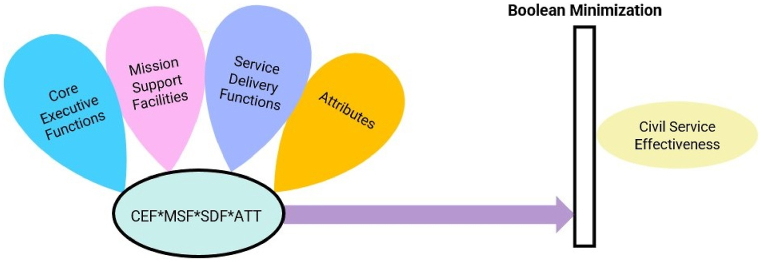
Venn diagram of the Configuration Model.

A conceptual model of this study is presented in Fig. 1 below based on the above propositions.

## 4 Methodological approach

### 4.1 Research data

The International CiSE Index represents a pioneering effort in evaluating global civil service performance, developed through a collaboration between the Blavatnik School of Government at Oxford University and the Institute for Government. This index provides a robust framework for assessing core executive functions, mission support, and service delivery across 38 nations, as detailed in its 2019 edition [66]. By utilizing a comprehensive set of indicators, the InCiSE Index enables aggregated and disaggregated evaluations of civil service effectiveness, focusing on central government functions [28]. The index serves dual purposes: it acts as a tool for performance enhancement, allowing civil service leaders to identify and learn from exemplary practices, and as a mechanism for accountability, offering a rigorous assessment of civil service performance for citizens and policymakers. Through its detailed methodology, the InCiSE Index aims to drive improvements in civil service performance, contributing to better governance and prosperity worldwide [28,67].

### 4.2 Data analysis technique

#### 4.2.1 Fuzzy-set qualitative comparative analysis (fsQCA)

The fsQCA methodology, pioneered by Charles Ragin in 1987 and further elaborated in 2009, leverages Boolean logic to explore theoretical relationships within social science models. It identifies logical linkages between desired outcomes and specific combinations of causal conditions, enhancing the understanding of the factors leading to particular results. An extensive examination of attributes and phenomena connected to every causal configuration in the model is what FsQCA does, expanding upon the body of research evidence [68]. A comprehensive exploration of the fundamentals of fsQCA is effectively guided by the insights of Pappas and Woodside [69]. FsQCA serves as a tool to examine how different combinations of conditions may lead to a particular outcome, effectively identifying which specific causal factors are essential or play a significant role in bringing about the observed result [70]. In addition, the use of ANN integrated with fsQCA likely stems from the complexity and size of the dataset, as well as the need for high predictive accuracy. While fsQCA is well-suited for identifying causal configurations and managing complex causal relationships with multiple pathways to an outcome, ANN excels in modeling non-linear relationships and recognizing patterns within datasets [71]. This methodological choice reflects a focus on predictive power and the ability to handle intricate data interdependencies, making ANN more appropriate for research that requires detailed pattern recognition and robust prediction capabilities.

FsQCA stands out for its ability to uncover asymmetric relationships and identify connections in studied cases that conventional statistical methods often overlook due to their reliance on statistical significance. Unlike traditional approaches that sever relationships with high p-values (e.g., p > 0.05) as potentially random, fsQCA considers case studies, enabling examining small sample sizes without the strict need for statistical significance. This approach, advocated by Woodside [72] and further supported by Thomann and Maggetti [73], Fiss [74] and mentioned by Roger-Monzó and Castelló-Sirvent [75], emphasizes understanding the nuances of causal relationships within the phenomenon being studied. By prioritizing consistency and coverage over significance, fsQCA can reveal causal connections in one or multiple cases through specific combinations of conditions. This method provides a unique perspective on causal analysis, especially in instances where traditional inferential statistics, such as regression analysis, would find a single case to be of low significance [73]. Through fsQCA, researchers can explore and explain unique cases, gaining new insights into reality by examining case studies and asymmetric relationships, thus offering a richer understanding of the phenomena at hand.

Bridging the gap between statistical and configurational methodologies illuminates aspects of reality that might remain obscured under the stringent criteria of inferential statistics. While each approach offers a distinct path to unraveling research questions, a developing trend is emerging that advocates for their integration [69]. This innovative approach seeks to enrich the empirical knowledge pool, merging the strengths of both techniques to forge more robust theories for intricate phenomena. By synthesizing these methodologies, researchers are poised to fill lingering gaps in academic literature, advancing our understanding of complex realities with unprecedented clarity and depth.

Using diverse logical frameworks, the study's comparative analysis of CSE unveils the determinants behind their thematic focus. The research employs iterative, case-oriented methods to enhance methodological rigor, in contrast to the emphasized meticulous approach [76,77]. It identifies two key elements: the outcome, defined as CSE, and the explanatory variables (CEF, MSF, SDF, ATT) influencing this outcome. The proposed causal model for examining the Civil Services Effectiveness is succinctly expressed as:

CSE = f (CEF, MSF, SDF, ATT)

In the fsQCA approach, calibrating the model's conditions for categorization is a crucial initial step. This process involves converting the original data into scores that align with fuzzy set theory, which assigns values from 0 to 1 to reflect degrees of set membership [72,78]. This calibration facilitates the identification of how closely observed cases align with a fuzzy set for each condition, guided by three critical thresholds: full membership (>0.95), maximum ambiguity (=0.5), and non-membership (<0.05). Within the fsQCA framework, cases with a membership score of 0.50 are excluded from analysis, as this midpoint score does not signify the presence or absence of a condition necessary for the occurrence of the studied phenomenon. Determining the three cutoff points relies on thoroughly understanding CSE and the specific countries under examination. This approach eschews the use of arbitrary benchmarks from prior studies, ensuring that the cutoff points for each attribute are appropriately tailored [79]. The second phase of the fsQCA technique unfolds with creating a ‘truth table'.This table compiles all conceivable combinations of the conditions in the model to elucidate the outcome [79]. Here, a decisive cutoff threshold of 0.80 is set to sift through these combinations, prioritizing those ranging from the cutoff point up to 1. The designs that adequately account for the subject matter concentration within CSE are distinguished from those that do not by this threshold, which serves as an essential filter. Furthermore, the chosen benchmark for examining causal links within CSE adopts a consistency level of 0.8346, surpassing the minimum threshold needed, ensuring a robust analysis of sufficient configurations. In the third stage, the model's solution is developed, encompassing three varieties: “complex,” “parsimonious,” and “intermediate.” Among these, the intermediate solution is deemed superior to both the complex and parsimonious solutions, as it retains all essential conditions without elimination [78]. Consequently, the intermediate solution for analyzing and interpreting the model's results is recommended.

#### 4.2.2 ANN analysis

Aw, Zha [80] stated that Fuzzy-set QCA was employed to discern the specific conditions—namely, core executive function, mission support facilities, service delivery functions, and attributes—that significantly contribute to CSE. These identified conditions were subsequently utilized as input variables for ANN analysis. As Hew, Badaruddin [81] posited, ANN is a computational model inspired by the neural networks of the human brain, capable of learning and acquiring new knowledge through experience. This adaptability of ANN allows for enhancing its predictive accuracy through training [82]. A conventional multi-layer perceptron ANN architecture includes input, hidden, and output layers, as detailed by Hew, Leong [83]. These layers are composed of neuron nodes interconnected by synaptic weights, which undergo modifications during the learning process through a non-linear activation function to optimize the network's performance towards predefined objectives [84]. Consequently, the integration of a more profound learning process through the inclusion of an adequately structured hidden layer, as recommended by Leong, Hew [71], is anticipated to refine the predictive capability of the ANN model. This methodological approach underscores the utility of leveraging fsQCA-derived conditions as foundational inputs for ANN analysis to enhance the predictive modeling of CSE.

In the execution of the ANN analysis within this study, the feed-forward back-propagation algorithm was selected for its efficiency in error reduction throughout the deep learning phase, as highlighted by Ooi, Hew [85]. To address overfitting, recommended by Leong, Hew [82] a 10-fold cross-validation technique was employed, effectively partitioning the data into a training-to-testing ratio of 9:1. Furthermore, the choice of a hyperbolic tangent as the non-linear activation function enhances the model's capability to manage complex relationships between inputs and outputs, while the determination of the optimal number of neurons within the hidden layer was entrusted to the ANN's analytical processes [86]. The evaluation of predicted accuracy, which is crucial to assessing the effectiveness of the ANN model, uses the root mean squared error (RMSE), an essential statistic derived from the sum of square errors (SSE). According to Ooi, Lee [87], a lower RMSE value indicates superior predictive precision. Complementing this measure, the goodness of fit (GoF) index (R^2^), as proposed by Leong, Hew [88], offers additional insight into the model's accuracy in reflecting the observed data. Further analysis of the model's effectiveness involves a sensitivity analysis, which ranks the input neurons based on their influence on the output neurons, thus elucidating their relative significance in the model [84]. Given the diverse nature of the InCiSE indicators, the construction of the ANN model precisely accommodates these distinct types, ensuring an insightful and accurate representation of CSE within the analytical framework.

## 5 Results and interpretation

### 5.1 Descriptive analysis

The descriptive statistics analysis (Table 1) facilitates a nuanced exploration of the relationships among various variables critical to understanding CSE. These variables encompass core executive functions (CEF), mission support facilities (MSF), service delivery functions (SDF), and attributes (ATT), and all posited as likely independent variables that elucidate variations in civil service effectiveness (CSE), the dependent variable under scrutiny. Notably, CSE is not subject to the variance inflation factor (VIF) evaluation because it is the dependent variable, contrasting with the independent variables where VIF is used to assess the level of multicollinearity in the regression model. The VIF values, ranging from 1.55 to 2.60 for the independent variables, suggest a low probability of multicollinearity, thereby affirming the utility and distinctiveness of each variable in contributing valuable insights without significant redundancy [89]. As VIF exceeding 5 or 10 signals problematic multicollinearity, the current study's VIF metrics indicate a satisfactory level of multicollinearity.

**Table 1:** Descriptive analysis.

**Variables**	**VIF**	**Mean**	**Std. Dev.**	**Minimum**	**Maximum**
CSE	–	0.5160526	0.3219966	0.03	0.97
CEF	2.60	0.5723684	0.2349629	0.19	0.95
MSF	2.00	0.5815789	0.2447504	0.11	0.94
SDF	1.55	0.6171053	0.2561446	0.09	0.97
ATT	2.00	0.5986842	0.2259969	0.14	0.93

Descriptive measures such as mean values, standard deviations, and range (minimum and maximum values) offer additional insight into the dataset. The mean values, indicating the average scores across variables, shed light on the typical manifestation of each factor within the population studied. For instance, the CSE's mean score of 0.516 suggests a moderate level of CSE in the sample. The standard deviation points to the degree of dispersion around the mean, with the dataset reflecting moderate variability across variables. This variability is further contextualized through the analysis of minimum and maximum values, elucidating the breadth of CSE observed within the sample, implied by CSE's range from 0.03 to 0.97. This thorough descriptive statistical analysis highlights the inherent diversity and potential improvement areas across various categories, illuminating the complex landscape of CSE. By examining the average performance and variability within each domain, the study provides a foundation for in-depth understanding and targeted improvements in civil service effectiveness, thereby contributing to optimizing public sector performance.

### 5.2 Correlation analysis

Analyzing correlation coefficients in Table 2 within the constructed matrix provides insights into the relationships among constructs pivotal to understanding CSE. These constructs include CSE, CEF, MSF, SDF, and ATT, with each correlation coefficient (r) ranging from −1 to 1. A value of 1 denotes a perfect positive relationship, −1 is an ideal negative, and 0 indicates no correlation. Notably, the correlation between CiSE and CEF is r = 0.9144, delineating a strong positive association. It suggests a direct correlation between the enhancement of core executive functions and the augmentation of civil service effectiveness, highlighting the pivotal role of CEF in civil service optimization. In a similar vein, there is a strong positive association (r = 0.7803) between CSE and MSF, highlighting the importance of mission support facilities in enhancing civil service performance. The association between CSE and SDF, marked at r = 0.7165, suggests a substantial positive correlation, albeit slightly less pronounced than that between CSE and CEF, implying that while SDF plays a crucial role in enhancing CSE, its impact may be moderated by other variables. Likewise, the strong positive correlation (r = 0.8488) that exists between CSE and ATT highlights the crucial role that organizational attributes play in determining the effectiveness of the civil service and emphasizes the need for creating supportive organizational environments. Interrelations among CEF, MSF, SDF, and ATT reveal varying degrees of correlation strength, with CEF-MSF (r = 0.6838), CEF-SDF (r = 0.5702), and CEF-ATT (r = 0.6721) indicating a foundational interdependence of core executive functions on mission support, service delivery, and attributes, respectively. Conversely, the correlations among MSF-SDF (r = 0.4537), MSF-ATT (r = 0.5887), and SDF-ATT (r = 0.5051) suggest moderate positive relationships, highlighting distinct yet interconnected impacts on CSE. This comprehensive correlation analysis underscores various constructs' integral and interconnected nature in enhancing civil service effectiveness. Hence, the statistically substantial relationships, especially between CSE and the corresponding components, highlight the need for a holistic approach to improving the civil service. In order to effectively maximize civil service performance, such an approach requires simultaneous direct attention to organizational attributes, service delivery functions, support facilities, and core executive functions. This interpretation allows for a nuanced understanding of the dynamics within civil service and highlights potential levers for enhancing effectiveness.

**Table 2:** Correlation matrix analysis.

**Construct**	**CiSE**	**CEF**	**MSF**	**SDF**	**ATT**
CSE	1.0000				
CEF	0.9144	1.0000			
MSF	0.7803	0.6838	1.0000		
SDF	0.7165	0.5702	0.4537	1.0000	
ATT	0.8488	0.6721	0.5887	0.5051	1.0000

### 5.3 Analysis results from fuzzy-set QCA

#### 5.3.1 Necessary condition analysis (NCA)

The NCA, as provided in Table 3, reveals a comprehensive and detailed understanding of the components that contribute to CSE. This study evaluates conditions such as CEF, MSF, SDF, and ATT for their indispensability and impact on CiSE. The study employs two metrics: consistency and coverage. Consistency measures the degree to which a specific condition is always present when the outcome occurs. It is akin to a conditional probability but within the context of QCA. A higher consistency score suggests that the condition is nearly always necessary for the outcome. Coverage assesses the proportion of instances of the outcome that the condition can explain. It reflects the condition's empirical importance or relevance across the cases studied [90].

**Table 3:** Necessary conditions for research constructs.

	Outcome Variable:	Civil Service Effectiveness (CSE)	
Listed Variables	Condition Tested	Consistency	Coverage
CRM, FFM, REG, POL	CEF	0.934727	0.842759
	∼CEF	0.442121	0.533539
HRM, PRO	MSF	0.902091	0.800452
	∼MSF	0.445691	0.549686
DIG, TAX	SDF	0.911269	0.762047
	∼SDF	0.399286	0.538144
CAP, INC, INT, OPN	ATT	0.939317	0.809670
	∼ATT	0.431412	0.554754

CEF: Core executive functions; MSF: Mission support facilities; SDF: Service delivery functions; ATT: Attributes; CAP: Capabilities; CRM: Crisis and risk management; DIG: Digital services; FFM: Fiscal and financial Management; HRM: Human resources management; INC: Inclusiveness; INT: Integrity; OPN: Openness; POL: Policymaking; PRO: Procurement; REG: Regulation; TAX: Tax administration; Following the terminology, the symbol (∼) represents the negation of the characteristic.

CEF is highly significant for CSE, evidenced by a consistency of 0.934727 and coverage of 0.842759, indicating that efficient core functions are almost invariably needed for enriched civil service performance. Conversely, the absence of CEF shows much lower consistency (0.442121) and moderate coverage (0.533539), indicating that lack of CEF severely reduces the likelihood of achieving CSE. MSF is identified as necessary, consistent with 0.902091 and coverage of 0.800452, affirming that robust mission support facilities correlate closely with higher CSE, albeit slightly less so than CEF. The reduced consistency (0.445691) and moderate coverage (0.549686) without MSF further attest to its necessity. In addition, SDF demonstrates a substantial necessity for CiSE, with a consistency of 0.911269 and coverage of 0.762047. The notable decrease in both metrics in the absence of SDF emphasizes the critical function of service delivery in CSE. Eventually, ATT, with the highest consistency (0.939317) and significant coverage (0.809670), underscores the importance of organizational attributes in achieving CiSE. The considerable reduction in consistency (0.431412) and coverage (0.554754) without ATT highlights the indispensable influence of these attributes on CSE. Hence, these results are remarkably close to the ground truth with Pappas and Bley [90], who stated the NCA substantiates that CEF, MSF, SDF, and ATT are integral and nearly indispensable for achieving civil service effectiveness. Their strong association with higher CSE levels, contrasted with the significant impact of their absence, delineates a multifaceted approach necessary for cultivating an effective civil service. This comprehensive analysis accentuates the imperative for a holistic strategy that addresses core functions, support mechanisms, service delivery, and organizational attributes to optimize civil service performance effectively.

#### 5.3.2 Develop truth tables and minimizing logic expressions

In this analysis phase, the fsQCA software version 3.1 generated a truth table from the calibrated dataset (Appendix A). This truth table comprises 2^n^ rows, where n represents the number of conditions evaluated. Consequently, each row corresponds to a unique permutation of the conditions. As an instance, with four conditions present, the truth table enumerates 16 distinct logical combinations. After formulating the truth table (referenced as Table 4), it is imperative to categorize it by applying specific thresholds for frequency and consistency. Table 4 illustrates the constructed truth table following these criteria.

**Table 4:** Truth table generating.

**CEF**	**MSF**	**SDF**	**ATT**	**Number**	**Cases**	**Raw consistency**	**PRI consistency**	**SYM consistency**
1	1	1	1	15 (42 %)	GBR, NZL, CAN, FIN, AUS, DNK. NOR, NLD, KOR, USA, EST, IRL, FRA, AUT, ESP	0.949662	0.905639	0.905639
0	0	0	0	6 (60 %)	SVK, BGR, HRV, ROU, GRC, HUN	0.558089	0.02864	0.028640
0	0	1	0	3 (68 %)	PRT, ISL, TUR	0.742353	0.102459	0.102459
1	1	1	0	3 (77 %)	LTU, JPN, ITA	0.860733	0.400901	0.413953
0	0	0	1	2 (82 %)	SVN, POL	0.672840	0.076655	0.076655
1	1	0	0	1 (85 %)	CZE	0.801258	0.229269	0.229269
1	0	1	0	1 (88 %)	MEX	0.831933	0.226519	0.241176
1	1	0	1	1 (91 %)	CHE	0.865705	0.584193	0.584193
0	0	1	1	1 (94 %)	LVA	0.827304	0.285024	0.285024
1	0	1	1	1 (97 %)	SWE	0.890890	0.624087	0.624088
0	1	1	1	1 (100 %)	BEL	0.884774	0.502223	0.515982
1	0	0	0	0 (100 %)	–			
0	1	0	0	0 (100 %)	–			
0	1	1	0	0 (100 %)	–			
1	0	0	1	0 (100 %)	–			
0	1	0	1	0 (100 %)	–			

The implementation of a periodic breakpoint is crucial to ensure that the collection of research data remains manageable and relevant, particularly in the context of fsQCA. For studies involving medium to small sample sizes, i.e., fewer than 120 cases, the recommended frequency cutoff is established at 1 to maintain the inclusivity of data points. Conversely, for analyses encompassing larger datasets, i.e., exceeding 120 cases, it is prudent to elevate the cutoff value above 1 to refine its relevance and manageability. With a sample size of 35 cases in the current study, we adhere to a frequency cutoff point of 1, aligning with the guidelines for smaller datasets. Furthermore, Brush, Guo [91] emphasize the potential pitfalls associated with setting a low consistency threshold, which may inadvertently allow for the inclusion of false-positive instances, thereby compromising the integrity of the analysis. Our study adopts a stringent consistency criterion to mitigate this risk, setting the threshold above 0.80. This threshold is not arbitrary but is informed by the specialized requirements of fuzzy-set analysis, ensuring that only those instances demonstrating a high degree of conformance to the hypothesized causal conditions are considered. This approach enhances the robustness of our findings and aligns with best practices in fsQCA methodology.

Table 5 unfolds the outcomes of the analytical study into the intricate factors fueling CSE. The analysis reveals that the consistency values, indicative of the reliability of causative factors in predicting CSE, range from 0.80 to 0.95 for both the presence and absence of these factors. Similarly, consistency values fluctuate between 0.86 and 0.97 for negating CSE, depicting intricate connections instead of a simple cause-and-effect progression. According to Schneider and Wagemann [92], none of the causative factors individually qualifies as strictly necessary for achieving either high or low levels of CSE, given that all surpass the threshold of 0.9. It indicates adaptability and potential synergy between CSE, ATT, SDF, MSF, and CEF causative factors. To further clarify the dynamics of CSE, the study employs a fuzzy set analysis, aiming to uncover sufficient combinations of causal conditions that account for variations in CSE. The results, as exhibited in Table 6, present the set-theoretic coherence outcomes for each causal combination and the aggregate explanation, with all values exceeding the critical threshold (>0.8) as per Pappas and Woodside [69]. In this context, ‘consistency’ pertains to the extent to which a causal nexus is consistently observed across different cases, whereas ‘coverage’ assesses the proportion of cases where a consistent causal pattern holds empirical significance. This analysis phase offers a nuanced understanding of the complex interplay between various conditions and their collective impact on CSE.

**Table 5:** Truth table after logical minimization.

**CEF**	**MSF**	**SDF**	**ATT**	**Number**	**CiSE**	**Member Cases**	**Raw consistency**	**PRI consistency**	**SYM consistency**
1	1	1	1	15 (42 %)	1	GBR, NZL, CAN, FIN, AUS, DNK. NOR, NLD, KOR, USA, EST, IRL, FRA, AUT, ESP	0.949662	0.905639	0.905639
0	0	0	0	6(60 %)	0	SVK, BGR, HRV, ROU, GRC, HUN	0.558089	0.028640	0.028640
1	1	1	0	3(68 %)	1	PRT, ISL, TUR	0.860733	0.400901	0.413953
0	0	1	0	3(77 %)	0	LTU, JPN, ITA	0.742353	0.102459	0.102459
0	0	0	1	2(82 %)	0	SVN, POL	0.672840	0.076655	0.076655
1	0	1	1	1(85 %)	1	CZE	0.890890	0.624087	0.624088
0	1	1	1	1(88 %)	1	MEX	0.884774	0.502223	0.515982
1	1	0	1	1(91 %)	1	CHE	0.865705	0.584193	0.584193
1	0	1	0	1(94 %)	1	LVA	0.831933	0.226519	0.241176
0	0	1	1	1 (97 %)	1	SWE	0.827304	0.285024	0.285024
1	1	0	0	1(100 %)	1	BEL	0.801258	0.229269	0.229269

**Table 6:** Configurational paths of intermediate solution using algorithm of Quine-McCluskey.

Path of Solutions	Raw Coverage	Unique Coverage	Consistency
Model: CSE = ƒ (CEF∗MSF∗SDF∗ATT)			

Frequency cutoff: 1 Consistency cutoff: 0.801258			
CEF∗MSF	0.866904	0.0586436	0.883576
CEF∗SDF	0.862825	0.0081591	0.882169
SDF∗ATT	0.874554	0.0392656	0.886763

Solution consistency	0.822707		
Solution coverage	0.960734		

#### 5.3.3 Fuzzy-set QCA findings

As shown in Table 7, the analysis delineates three distinct pathways that can lead to CSE in the framework of the Fuzzy-set QCA technique into CSE. The notation within the table employs black circles (●) to signify the presence of a condition, cross marks (⊗) to indicate blank cells to represent a ‘do not care’ condition, implying the indifference towards the presence or not required of a condition in that particular configurational analysis. Our findings, encapsulated in Table 6, reveal an overall solution consistency of 0.822707 and a coverage of 0.960734, suggesting that the three identified causal configurations possess empirical significance and collectively offer a comprehensive understanding of the pathways to CSE. Notably, the analysis underscores the premise that no single condition is paramount in isolation; rather, the synergistic interaction within these configurational sets underpins CSE. Coverage, as articulated by Rihoux and Ragin [93], is a metric to evaluate the empirical relevance or significance of the underlying conditions or combinations thereof. It measures the extent to which the observed outcomes across cases can be attributed to either a single condition or a nexus of conditions, thus emphasizing the empirical significance of the identified configurations in explaining CSE. This approach facilitates a nuanced comprehension of the multifaceted and interconnected conditions that contribute to achieving optimal civil service performance, highlighting the importance of considering complex causalities in assessing CSE.

**Table 7:** Multifactor combination analysis of conditions for the civil service effectiveness.

**Configuration**	**Solution**		
	**1 (CEF∗MSF)**	**2 (CEF∗SDF)**	**3 (SDF∗ATT)**
Core Executive Functions	●	●	⊗
Mission Support Facilities	●	⊗	⊗
Service Delivery Functions	⊗	●	●
Attributes	⊗	⊗	●
Consistency	0.883576	0.882169	0.886763
Raw Coverage	0.866904	0.862825	0.874554
Unique Coverage	0.0586436	0.0081591	0.0392656

Overall Solution Consistency	0.822707		
Overall Solution Coverage	0.960734		

Note: Black circles (●) condition indicate that the variables exist and has a high effect; the cross-circle with the ‘X’ (⊗) condition indicates that the impact of the variable is not required.

Configuration 1 (CEF∗MSF) elucidates a synergistic interaction between core executive functions and mission support facilities, accentuating their collective importance in augmenting the efficacy of civil service systems. The manifestation of both conditions, as denoted by the presence of black circles (●), indicates their indispensability, with a consistency measure of 0.883576 and a raw coverage of 0.866904. This configuration emphasizes the pivotal role of strategic governance elements, including policymaking, fiscal and financial management, regulatory frameworks, and crisis management, alongside essential mission support operations such as procurement and HR management. These components are integral to creating a civil service framework that is resilient and highly effective. The unique coverage value of 0.0586436 further delineates the percentage of cases clearly explained by this configuration, underscoring the synergy between core executive functions and mission support facilities as crucial for realizing superior civil service performance. This pathway advocates for a concentrated focus on these areas as essential for promoting public sector efficacy by offering a more sophisticated understanding of the routes via which the integration of CEF and MSF serves as a cornerstone for improving civil service effectiveness.

Configuration 2 (CEF∗SDF) delineates an integrated model that amalgamates core executive functions with service delivery functions, spotlighting the imperative synergy between strategic governance processes and the execution of services. This configuration underscores the significance of harmonizing elements such as policymaking, fiscal and financial oversight, regulatory frameworks, crisis management, tax administration, and digital service provisioning to bolster civil service effectiveness. It posits a model of civil service wherein strategic executive functions and service delivery mechanisms are interwoven and mutually reinforcing rather than operating in isolation. This paradigm asserts that the efficacy and impact of civil services are optimized when the executive functions, which delineate strategic orientations, policy development, and overarching governance, are intricately inked with the operational tasks that deliver services directly to citizens. In line with the fundamentals of Configuration 1, 0.882169 for consistency and 0.862825 for raw coverage verify that these conditions are essential.

Nonetheless, the unique coverage for this configuration is modest at 0.008159, suggesting its necessity but highlighting its role in explaining a more petite, unique segment of the CSE index variance. Moreover, this configuration acknowledges that CSE transcends the mere quality of executive-level policy and decision-making. It equally hinges on the operational efficiency and responsiveness of the service delivery mechanisms. Using this lens, the configuration supports civil service roles that ensure the delivery of services aligns with and fulfils strategic objectives, leading to increased operational effectiveness and impact. This analytical perspective emphasizes the importance of strategic planning and operational execution in pursuing elevated CSE.

Configuration 3 (SDF∗ATT) emerges as a critical pathway, elucidating the synergistic relationship between service delivery functions and attributes. This configuration posits that integrating attributes within the service delivery mechanisms substantially influences CSE. These attributes—integrity, openness, capabilities, and inclusiveness, among others—serve as the foundational pillars that ensure services are not only accessible but also align with the highest standards of public expectation. The configuration advocates for a paradigm shift towards user-centric service delivery routes, emphasizing creating a positive experience for service recipients as a cornerstone for enhancing overall service effectiveness and public satisfaction. Furthermore, this path highlights that the intrinsic qualities or features of the service delivery processes hold equivalent, if not more sumptuous, significance in determining the effectiveness of civil services compared to those rendered. With a consistency score of 0.886763 and a raw coverage of 0.874554, Configuration 3 underscores the robust influence of service delivery and organizational attributes on CSE. Notably, the unique coverage of 0.0392656 indicates a moderate unique explanatory power in accounting for variances in CSE, underscoring the distinct impact of this configuration relative to others. This approach fosters continuous improvement innovation and reinforces the importance of integrity, transparency, and inclusiveness in building trust and credibility with the public. In essence, configuration 3 articulates a compelling argument that the efficacy of civil services transcends the mere provision of services; it is equally contingent upon how these services are delivered. This configuration, therefore, lays the groundwork for a comprehensive understanding of CSE, advocating for a holistic approach that integrates positive service attributes into the fabric of service delivery mechanisms to achieve optimal effectiveness and elevate public satisfaction.

**Fig. 2:**
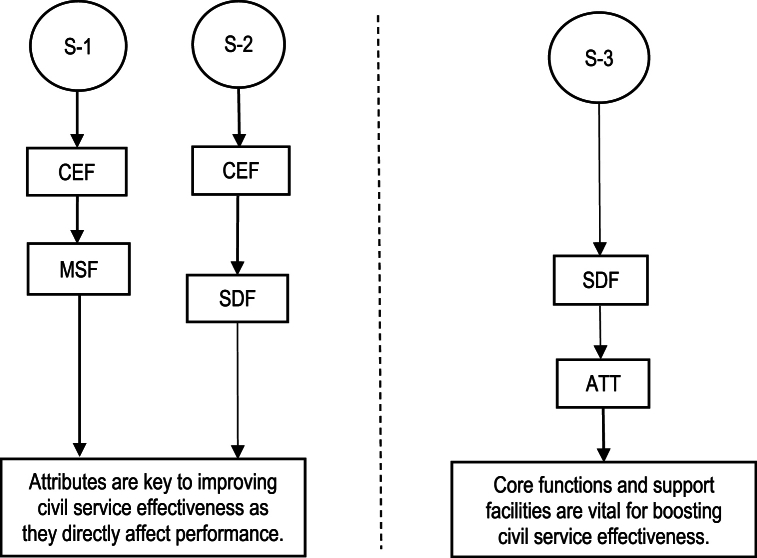
Configurational pathways to variables are represented visually.

The aggregate solution consistency of 0.822707 and coverage of 0.960734 for the identified configurations substantiate our study's methodological rigor and comprehensive scope. These metrics reflect that CSE cannot be attributed to a singular factor but emerges from the synergistic interplay among multiple conditions. The configurations delineated in this study underscore the inherent complexity in achieving optimal CSE and offer detailed insights into the specific combinations of conditions that civil service organizations could strategically focus on to augment their operational efficacy. This analysis contributes a sophisticated framework for understanding the roles played by CEF, MSF, SDF, and ATT in cultivating CSE. It extends beyond identifying influential factors, offering a blueprint for how these elements can be integrated and leveraged to advance civil service performance. Such insights are critical to policymakers and practitioners dedicated to enhancing civil services, presenting a nuanced approach to implementing reforms aimed at achieving intensified efficiency and effectiveness within the public sector. Fig. 2 depicts graphical representations of alternative solutions.

Table 8 elucidates the causal configurations affecting civil service outcomes across 25 countries, employing symbols '✓' and '⊗' to indicate the presence or absence of specific configurations involving CEF, MSF, SDF, and ATT. The analysis categorizes the findings into common configurations, unique deviations, regional variations, and specific observations. In common configurations, most countries (17 out of 25) display a pattern where CEF, in conjunction with MSF or SDF, and the interplay between SDF and ATT are consistently present, suggesting a prevalent model that synergizes these factors to impact civil service effectiveness. Furthermore, unique deviations in a subset of countries, including Switzerland, Czechia, Mexico, Sweden, Belgium, Latvia, and Germany, present unique configurations, diverging from the common pattern. Switzerland and Czechia are notably absent in two configurations, indicating alternative mechanisms at play. Likewise, regional variations, while not explicitly detailed, and the presence of common configurations and unique deviations across countries from different regions—Europe, North America, and Asia—may reflect distinct regional governance models, policy priorities, or administrative cultures.

**Table 8:** Casual configurations proposed by the model.

**SI**	**Country**	**Casual configurations**		
		**CEF∗MSF**	**CEF∗SDF**	**SDF∗ATT**
1	United Kingdom	✓	✓	✓
2	South Korea	✓	✓	✓
3	New Zealand	✓	✓	✓
4	Canada	✓	✓	✓
5	Australia	✓	✓	✓
6	United States	✓	✓	✓
7	Switzerland	✓	⊗	⊗
8	Denmark	✓	✓	✓
9	Norway	✓	✓	✓
10	Netherlands	✓	✓	✓
11	Finland	✓	✓	✓
12	France	✓	✓	✓
13	Estonia	✓	✓	✓
14	Lithuania	✓	✓	⊗
15	Japan	✓	✓	⊗
16	Italy	✓	✓	⊗
17	Ireland	✓	✓	✓
18	Czechia	✓	⊗	⊗
19	Austria	✓	✓	✓
20	Spain	✓	✓	✓
21	Mexico	⊗	✓	⊗
22	Sweden	⊗	✓	✓
23	Belgium	⊗	⊗	✓
24	Latvia	⊗	⊗	✓
25	Germany	⊗	⊗	✓

Finally, specific observations show that countries like Mexico, Sweden, Belgium, Latvia, and Germany, which are absent in the CEF∗MSF configuration but present in others, might prioritize aspects such as SDF or ATT over traditional core executive functions. The above map in the table reveals a predominant trend where a synergistic interaction between CEF, MSF, SDF, and ATT is common across a majority of the examined countries, with notable exceptions reflecting unique institutional arrangements or focus areas within their civil service systems.

**Fig. 3:**
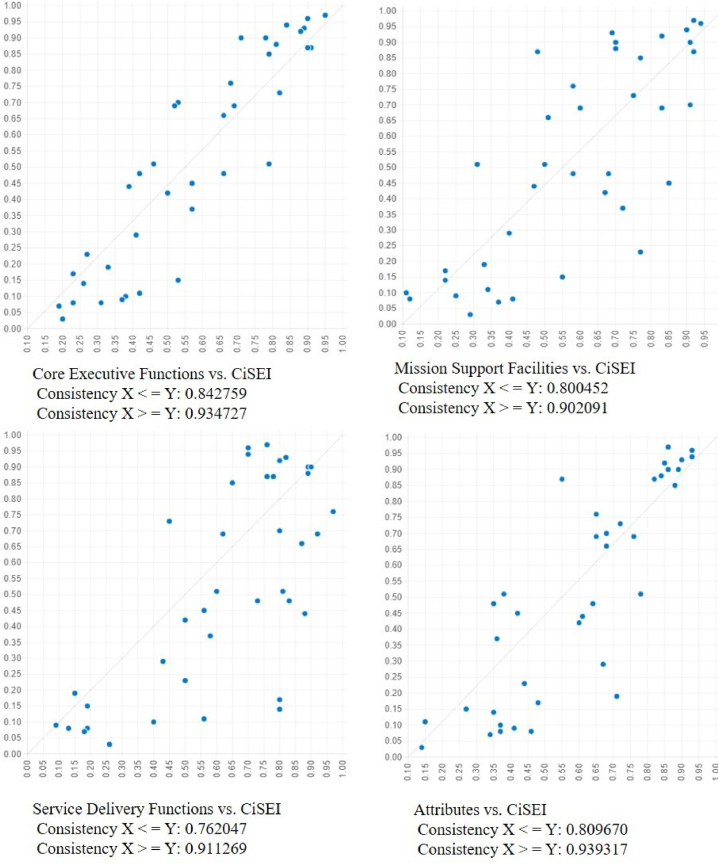
XY Plot charts by conditions and results.

We identify two distinct CSE topologies by analyzing intermediate solutions and subsequent discussions, as delineated in Table 9. These topologies necessitate the enduring presence of attributes, core functions, and support service facilities to sustain CSE. Fig. 3 graphically displays these outcomes and their correlating variables in XY plots, facilitating an analysis of the conditions under which specific variables—such as core executive functions, mission support facilities, and attributes—exhibit high, low, absent, or no correlation with the outcome across a variety of cases with differing variable configurations.

**Table 9:** Pathways leading to civil service effectiveness topologies.

Label	Definition	Key facts
Attributes underpin civil service effectiveness by shaping behavior and decision-making (S-1 and S-2)	1 High-level mission support facilities and key executive functions combined. 2 High-core executive functions coupled with high-service delivery facilities.	1) Effective civil services necessitate the seamless integration of support for the overarching mission with executive decision-making, ensuring that strategic planning and support departments are closely aligned with executive functions to achieve the organization's objectives. 2) A strong link between executive decision-making and service delivery is essential to ensure that high-level decisions are based on practical service aspects to enhance the efficiency and responsiveness of civil service operations.
Core functions and support facilities are vital for supplying the infrastructure and resources (S-3)	1 Enhanced service delivery functions combined with attributes.	1) The enhancement of civil service effectiveness and efficiency is significantly facilitated through the strategic integration of core functions and support facilities with essential attributes, highlighting that mere infrastructure and resources are inadequate for achieving optimal performance.

### 5.4 ANN analysis

The fsQCA analytical approach exposes distinct configurational pathways conceptualized as analogous to neural networks within the ANN framework [94]. This investigation adopts a k-fold cross-validation strategy, specifically utilizing ten folds to mitigate the risk of overfitting. This approach allocates 90 % of the dataset for training purposes and the remaining 10 % for validation, adhering to the methodology advocated by Leong, Hew [82]. The layout of the resulting ANN model comprises an input layer, integrating four key dimensions of civil service effectiveness, and an output layer designated for CSE outcomes. Table 10 presents the root mean square error (RMSE) metrics for the model's training and validation phases, offering critical insights into its predictive fidelity. Furthermore, Fig. 5 delineates that the average RMSE values range from 0.2 to 0.5 across both datasets, which indicates the ANN model's precision and robustness in encapsulating the dynamics between the predictor variables and CiSE [95]. The RMSEA indices for training and testing datasets corroborate the significant influence of the predictor variables on CiSE, with all values remaining below the threshold of 0.50 and demonstrating proximity to one another, affirming the model's accuracy and reliability.

**Table 10:** ANN-RMSE values.

ANN	**Training**			**Testing**			**Total Sample**s
	N	SSE	RMSE	N	SSE	RMSE	
ANN1	33	0.037	0.033485	5	0.005	0.031623	38
ANN2	34	0.024	0.026568	4	0.001	0.015811	38
ANN3	31	0.014	0.021251	7	0.006	0.029277	38
ANN4	32	0.019	0.024367	6	0.001	0.01291	38
ANN5	35	0.097	0.052644	3	0.002	0.02582	38
ANN6	34	0.048	0.037573	4	0.004	0.031623	38
ANN7	35	0.024	0.026186	3	0.002	0.02582	38
ANN8	33	0.046	0.037335	5	0.01	0.044721	38
ANN9	34	0.085	0.050000	4	0.001	0.015811	38
ANN10	37	0.057	0.03925	1	1.2E-07	0.000347	38

Mean		0.0451	0.034866		0.0032	0.023376	
SD		0.026535	0.010097		0.002926	0.011801	

Notes: RMSE = √ (1*/*n) × SSE and Leong et al. (2020) define the goodness-of-fit index (R^2^) as 1 minus the ratio of the average Root Mean Square Error (RMSE) during testing to the average Sum of Squared Errors (SSE) during testing.

Sensitivity analysis quantifies normalized importance by computing the ratio of each input's relative significance, subsequently expressed as a percentage [96,97]. This research quantifies the impact of variations in independent variables on the dependent variable. These crucial factors were hierarchically classified according to their normalized relative importance, as systematically documented in Table 11. Among the indicators for CSE, ‘core executive function’ emerged as the paramount predictor of CSE, achieving a normalized relative importance of 100 %. It was succeeded by ‘attributes’ and ‘service delivery function’. Conversely, ‘mission support facilities’ were identified as the dimension of least significance. Notably, these findings derived from the ANN analysis exhibit a strong unity with the outcomes delineated in the QCA results, as illustrated in Table 9, thereby attesting to the reliability of the model postulated in this study.

**Table 11:** Sensitivity analysis.

ANN	CEF	MSF	SDF	ATT
ANN1	1.0000	0.5107	0.6066	0.8491
ANN2	1.0000	0.4823	0.5762	0.8798
ANN3	1.0000	0.4226	0.5520	0.8380
ANN4	1.0000	0.4859	0.5894	0.9054
ANN5	1.0000	0.5486	0.7111	0.8663
ANN6	1.0000	0.4739	0.5378	0.9188
ANN7	1.0000	0.4801	0.5805	0.8046
ANN8	1.0000	0.4912	0.5099	0.8956
ANN9	1.0000	0.4175	0.6452	0.8081
ANN10	1.0000	0.4520	0.5266	0.8584

Average Importance	1.0000	0.4765	0.5835	0.8624
Normalized importance (%)	100 %	48 %	58 %	86 %
**Ranking**	**1**	**4**	**3**	**2**

**Fig. 4:**
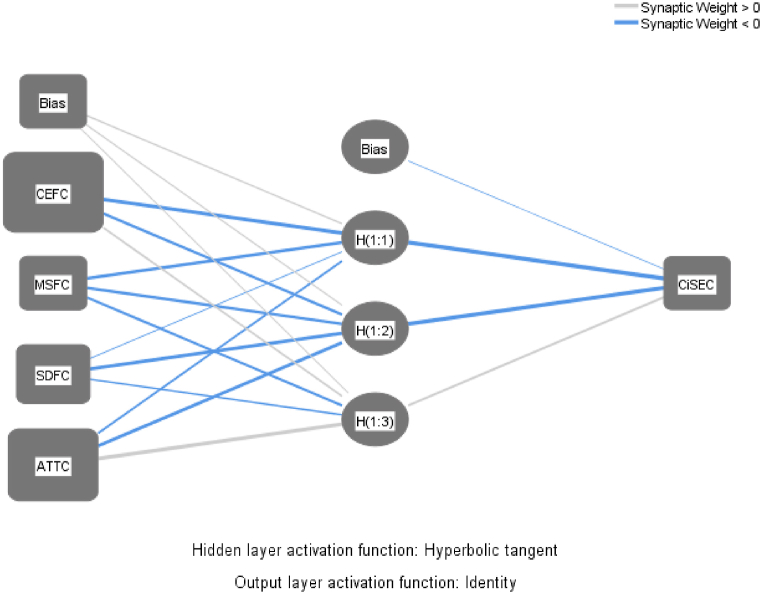
ANN diagram.

Furthermore, to evaluate the ANN model's predictive efficiency, a goodness-of-fit metric was calculated using the formula R^2^ = 1 - (RMSE/SSE) [82]. This computation reveals that the ANN model could accurately forecast 97.98 % of CiSE outcomes, demonstrating a significantly high level of predictive precision. The analysis via ANN produces a markedly superior R^2^ value compared to the results obtained through fsQCA analysis (R^2^ = 97.98 %, as presented in Table 7). This enhancement in predictive explanation for the endogenous variable, CiSE, can be attributed primarily to the ANN's adeptness at identifying and processing non-linear relationships among variables [84]. Fig. 4 visually details the constructed ANN model for this study, further illustrating its analytical framework.

**Fig. 5:**
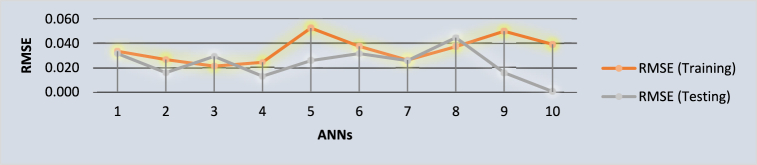
ANN diagram (training and test data).

## 6 Discussion

The research findings demonstrate positive correlations among CEF, MSF, SDF, and ATT. The application of ANN not only corroborates but also enhances the insights gained from fsQCA by enabling a detailed ranking of the dimensions influencing CSE [98]. FsQCA results indicate that each indicator of CSE is integral to at least one configuration for the effectiveness index assessed. Importantly, no single indicator is identified as either universally necessary or entirely irrelevant, which aligns with the principles of contingency theory [24,25], emphasizing that all elements are critical in determining CSE. The findings further reveal that there is no one-size-fits-all solution across different dimensions, suggesting variations in service regulations across countries lead to distinct behaviors and differences in their respective capacities and attributes. This diversity also means that configurations of indicators vary significantly across different dimensions and jurisdictions.

The study findings provide compelling evidence supporting the Configuration 1 (CEF∗MSF) model, highlighting noteworthy interaction between CEF and MSF as crucial for enhancing civil service effectiveness. With a consistency measure of 0.883576 and a raw coverage of 0.866904, integrating strategic governance elements with mission support operations emerges as both beneficial and essential for developing resilient and effective civil service frameworks. These results are consistent with the framework proposed by Steurer [99], who also emphasized integrating strategic and operational functions in public sector organizations, further validated by their similarly high consistency measures. The high consistency measures reported in their analysis further validate our findings on the pivotal importance of both core and support functions in maintaining robust and effective civil services. Additionally, the unique coverage value of 0.0586436 in this study underscores the specific contribution of the CEF∗MSF configuration to civil service performance, echoing the theoretical predictions of Brunet and Aubry [100] regarding the unique effects of integrated governance frameworks. The study reaffirms that aligning executive functions, such as policymaking, fiscal management, and crisis management, with mission support operations, including procurement and human resources, is critical for effective civil service. This finding is consistent with Meuleman [101], who demonstrated that strategic alignment across these functions leads to improved service delivery and organizational agility. However, the study also suggests avenues for further research, particularly the impact of cultural and institutional differences on the CEF∗MSF configuration, as noted by Haque [102]. Exploring these differences could provide insights into the adaptability of the CEF∗MSF model across various administrative and governance contexts.

In addition, this study demonstrates that CSE is markedly enhanced when CEF is well integrated with SDF, which supports the Configuration 2 (CEF∗SDF) model. This path implies that policies are well-planned and efficiently executed by successfully addressing practical requirements. Consistent with the theoretical propositions of Alcaide Muñoz, Alcaide Muñoz [103], who emphasized the synergy between strategic planning and operational execution, our findings reveal a high consistency score of 0.882169 and a raw coverage of 0.862825, underscoring the critical role of this model in optimizing civil service performance. However, the model's unique coverage of 0.008159 suggests that while it significantly contributes to explaining CSE, other factors or configurations may also play a role, as Ding and Riccucci [104] suggested. Echoing the work of Woo and Kim [41] on the vital interaction between high-level strategy and ground-level operations in improving public service responsiveness, our research highlights the importance of aligning policymaking, fiscal oversight, and service delivery mechanisms, including tax administration and digital services [32]. Moreover, drawing on Luna, Picazo-Vela [105], who discussed the role of digital platforms in enhancing service delivery, our study underscores the importance of digital services in connecting strategic objectives with service outcomes. This integrated approach improves service delivery, strengthens governance and increases citizen satisfaction and trust in government institutions, highlighting the need for a coherent strategy that aligns strategic governance with operational excellence.

The final configurational path in this study aligns with previous research, demonstrating that integrated service delivery mechanisms significantly enhance civil service effectiveness, particularly under Configuration 3 (SDF∗ATT). The study underscores the crucial role of integrity, openness, capabilities, and inclusiveness in shaping effective civil services, consistent with Van Dooren [66], who emphasized that these attributes ensure service delivery aligns with public expectations, fostering greater public trust and satisfaction. Our findings support the results of Witesman, Walters [106], highlighting the importance of adaptability in service delivery functions in response to changing public needs, which correlates with higher effectiveness ratings [62]. The unique coverage of 0.0392656 indicates that Configuration 3 has moderate explanatory power, suggesting the distinct impact of integrating service attributes. It is comparatively higher than the scores reported by Bonomi, Sarti [107] for other configurations, outlining the particular effect of integrating service attributes effectively within civil services. However, contrary to predictions by Idzi and Gomes [54], our findings did not support the significant impact of technology integration within SDF on civil service effectiveness, potentially due to regional disparities in technological adoption, as Saragih, Reyhani [52] noted. Notably, the ANN analysis verified previous findings, revealing through hierarchical classification based on the normalized relative importance that ‘core executive function’ is the topmost predictor of civil service effectiveness, with a normalized importance of 100 %. It was followed by ‘ATT’ and ‘SDF’, while ‘MSF’ were deemed the least significant. This consistency between ANN results and QCA outcomes underscores the robustness and validity of the model used. Moreover, the alignment illustrates the capability of ANN to effectively capture and analyze the complex interdependencies among the various factors [71] of CSE, thereby providing a comprehensive and theoretically aligned understanding within our study This alignment between ANN and fsQCA results demonstrates the robustness of the model and highlights the importance of a comprehensive approach to understanding CSE.

Table 9 elucidates the interconnected pathways that lead to CSE by integrating core propositions concerning executive functions, mission support facilities, service delivery, and essential attributes. The table underlines importance of aligning high-level mission support with executive decision-making (Proposition 1, Proposition 2) to ensure that strategic planning and operational support are closely linked, optimizing policymaking, fiscal management, and regulatory functions. It further highlights the critical role of service delivery functions (Proposition 3) in enhancing civil service performance when effectively coupled with executive functions, ensuring that practical service aspects inform high-level decisions. Additionally, the table emphasizes that embodying key attributes such as integrity, openness, capabilities, and inclusivity (Proposition 4) is indispensable for civil service effectiveness, demonstrating that these attributes, when integrated with service delivery, are fundamental to achieving optimal performance. Thus, Table 9 provides a comprehensive framework that synthesizes these propositions, illustrating how incorporating these elements contributes to the overall effectiveness of the civil service.

## 7 Implications and future work

### 7.1 Theoretical implications

This study makes a significant contribution to public sector effectiveness by examining the InCiSE reports across 35 countries and validating their relationships with CEF, MSF, SDF, and ATT, suggesting these to make them contextual. Grounded in contingency theory, this research provides a robust theoretical framework for an emerging area that highlights the critical influence of these elements on CSE and organizational effectiveness, which is contingent upon aligning internal components with specific demands of the external environment. The study also emphasizes the synergy between strategic objectives and practical operations in enhancing CSE by identifying key configurations that integrate strategic governance with operational support. Additionally, the study underscores the pivotal role of service attributes in building public confidence and trust. This theoretical perspective enriches the understanding of different configurations of CEF, MSF, SDF, and ATT, which can be optimized to improve civil service performance, offering valuable insights for both theoretical development and practical application in public administration and governance.

The research also advances the theoretical aspect of CSE by demonstrating the utility of the ANN approach in elucidating the relationships between these organizational elements and their impact on the international CSE rankings. The hierarchical classification of organizational components reveals that core executive functions and attributes are the primary drivers of civil service performance, with service delivery functions as supportive roles. These findings offer profound theoretical implications by enriching the discourse on the interactions between organizational components and external factors. This comprehensive approach deepens theoretical constructs within public administration and governance and provides a valuable framework for policymakers and practitioners aiming to optimize civil service operations in diverse contexts.

### 7.2 Methodological contribution

This research focuses on a hybrid quantitative-qualitative approach that addresses pertinent issues in public service. The methodological innovation of this study lies in its application of a dual analytical framework that integrates fsQCA and ANN techniques, providing a comprehensive analysis of CSE. This approach facilitates a nuanced understanding of the causal dynamics within the data, supported by a robust theoretical framework that draws extensively on prior case knowledge Rihoux [76]. The deployment of fsQCA has successfully identified three distinct causal configurations that elucidate the unique paths employed by civil servants focusing on international CSE issues. This study enhances analytical depth and robustness by integrating fsQCA and ANN, which are methodologies that complement each other effectively. FsQCA is instrumental in uncovering complex configurations among variables such as CEF, MSF, SDF, and ATT, thus capturing the intricate relationships influencing CSE. Concurrently, using ANN provides a detailed ranking of these dimensions, offering delicate insights into their relative significance. This methodological integration allows for a comprehensive exploration of the drivers of CSE, delivering substantial value to academic researchers and practitioners. Additionally, the blending of qualitative and quantitative approaches not only strengthens the credibility and validity of the research findings but also contributes to methodological innovations within the field of public administration. This synergy ensures the study's conclusions are well-founded and significantly enhances the scope of inquiry into public sector effectiveness.

### 7.3 Practical implications

This research has considerable practical applications since it offers practitioners and policymakers helpful information to improve the efficiency of public service operations. It identifies crucial configurations that optimize performance, such as integrating strategic governance elements with mission support operations and aligning high-level strategies with operational execution. These findings serve as a strategic guide for organizational improvement, enabling practitioners to develop more integrated governance frameworks responsive to the dynamic needs of citizens and government structures. Moreover, the research highlights the critical importance of prioritizing service attributes like integrity and openness, advocating for establishing a transparent and accountable organizational culture within civil services. This focus on ethical standards and open governance enhances service delivery and builds public trust. Furthermore, identifying the hierarchical significance of different organizational components allows policymakers to strategically allocate resources and prioritize initiatives that substantially impact civil service performance. This targeted approach facilitates the efficient use of resources and enhances the overall effectiveness and responsiveness of civil service institutions. Such insights are invaluable for driving systematic changes that align with both current needs and future demands within public administration.

### 7.4 Limitations and scope for further research

The limitations of the study are using fsQCA, the generalizability of these results across different governmental contexts or civil service systems is limited; the models tailored to specific CSE indicators might not fully encapsulate the diversity of administrative systems globally; identified ‘mission support facilities' as the least significant predictor could be an artifact of the particular dataset or methods used, potentially overlooking scenarios where these facilities play a critical role. The models suppose a static relationship between variables and outcomes, neglecting the potential dynamic changes in civil service functions influenced by evolving policies, societal shifts, and technological advancements. Furthermore, the findings of the study are context-specific that suggest cautious application.

Further investigation could enhance the existing models by implementing them across a more comprehensive array of governmental systems and evaluating their adaptability and resilience across diverse administrative structures. Longitudinal research would shed light on how the relationships between variables change over time, offering insights into the dynamic effects of changing variables on the effectiveness of the civil service. In line with this, adding predictors like technological advancements, public sentiment, and economic conditions could enrich this research understanding of the complex factors driving CSE. Besides, comparative analyses across different countries or administrative systems could shed light on the effects of cultural and institutional differences on civil service efficacy. Moreover, doing empirical research that explicitly examines the practical execution of these theoretical models could verify their relevance and provide valuable insights for policymakers seeking to improve civil service activities. By addressing these research avenues, future studies could deepen our comprehension of civil service effectiveness, paving the way for developing more responsive and adaptive administrative systems in Bangladesh and the globe.

## 8 Conclusion

This research explores the imperative of enhancing CSE amidst evolving governance challenges, globalized forces, and technological progress, underscoring civil services' central role in policy development and societal advancement. Utilizing a hybrid analytical framework that integrates Fuzzy-set QCA and ANN, the study dissects the intricate dynamics among organizational components essential for civil service efficacy. It highlights crucial setups, like MSF and governance and coordinating strategic plans with pragmatic implementation, that are essential for enhancing the effectiveness of the civil service. The findings explored significant interrelations between CEF, MSF, SDF, and ATT, advocating for a cohesive approach to strengthen civil service operations. The study also prioritizes CEF and ATT, suggesting they drive organizational performance. Theoretically, this work enriches understanding of civil service dynamics and organizational theory, corroborating contingency theory by stressing the need for organizational alignment with the external environment. Methodologically, the application of sQCA and ANN provides a nuanced, robust analysis framework, enriching the depth of the study. Practically, the research offers actionable insights for policymakers and practitioners focused on improving governance and public trust. By elucidating the complex interdependencies within civil service organizations, the study guides stakeholders in designing targeted interventions that promote transparency, accountability, and responsiveness, ultimately fostering a more effective and trustworthy public sector.

## CRediT authorship contribution statement

**Munshi Muhammad Abdul Kader Jilani:** Writing – original draft, Visualization, Validation, Software, Project administration, Methodology, Formal analysis, Conceptualization. **Md Mominur Rahman:** Writing – original draft, Validation, Resources, Data curation, Methodology, Formal analysis. **Md Abdul Latif:** Writing – review & editing, Visualization, Validation, Resources. **Nasim Ahmed:** Writing – review & editing, Visualization, Validation, Supervision, Methodology.

## Ethical statements

We employed open-source data from the Blavatnik School of Government at Oxford University and the Institute for Government in this study. The data utilized in this study is from their 2019 edition report, and we have accessed it in adherence to the open-access policy under which it was published. We have consistently upheld our commitment to academic integrity by duly acknowledging the original context and purpose for which the data was provided. Furthermore, we have provided proper attribution to the original authors and institutions, and the data has been exclusively utilized for academic research and analysis. No alterations have been made to the data that would distort the original findings.

## Data availability statement

The data supporting the findings of this study are available from the Blavatnik School of Government at Oxford University and the Institute for Government. This data can be openly accessed under the terms of the open-access policy. The 2019 edition report, which includes the data, can be obtained from the following link: (https://www.bsg.ox.ac.uk/about/partnerships/international-civil-service-effectiveness-index-2019). It is important to note that no new data was created in this study.

## Funding statement

This research was conducted without the support of any specific grants from funding agencies in the public, commercial, or not-for-profit sectors.

## Declaration of competing interest

The authors declare that they have no known competing financial interests or personal relationships that could have appeared to influence the work reported in this paper.

## Figures and Tables

**Table:** 

**1**	**2**	**3**	**4**	**5**	**6**	**7**
**Country Code**	**Country Name**	**CEF**	**MSF**	**SDF**	**ATT**	**CiSE**
		**0**–**1**	**0**–**1**	**0**–**1**	**0**–**1**	**0**–**1**
GBR	United Kingdom	0.95	0.92	0.76	0.86	0.97
NZL	New Zealand	0.90	0.94	0.70	0.93	0.96
CAN	Canada	0.84	0.90	0.70	0.93	0.94
FIN	Finland	0.89	0.69	0.82	0.90	0.93
AUS	Australia	0.88	0.83	0.80	0.85	0.92
DNK	Denmark	0.71	0.91	0.89	0.86	0.90
NOR	Norway	0.78	0.70	0.90	0.89	0.90
NLD	Netherlands	0.81	0.70	0.89	0.84	0.88
KOR	South Korea	0.91	0.92	0.76	0.55	0.87
SWE	Sweden	0.90	0.48	0.78	0.82	0.87
USA	United States	0.79	0.77	0.65	0.88	0.85
EST	Estonia	0.68	0.58	0.97	0.65	0.76
CHE	Switzerland	0.82	0.75	0.45	0.72	0.73
IRL	Ireland	0.53	0.91	0.80	0.68	0.70
FRA	France	0.69	0.83	0.62	0.65	0.69
AUT	Austria	0.52	0.60	0.92	0.76	0.69
ESP	Spain	0.66	0.51	0.87	0.68	0.66
MEX	Mexico	0.79	0.31	0.81	0.38	0.51
DEU	Germany	0.46	0.50	0.60	0.78	0.51
LTU	Lithuania	0.66	0.58	0.83	0.35	0.48
BEL	Belgium	0.42	0.68	0.73	0.64	0.48
JPN	Japan	0.57	0.85	0.56	0.42	0.45
LVA	Latvia	0.39	0.47	0.88	0.61	0.44
CHL	Chile	0.50	0.67	0.50	0.60	0.42
ITA	Italy	0.57	0.72	0.58	0.36	0.37
SVN	Slovenia	0.41	0.40	0.43	0.67	0.29
ISR	Israel	0.27	0.77	0.50	0.44	0.23
POL	Poland	0.33	0.33	0.15	0.71	0.19
PRT	Portugal	0.23	0.22	0.80	0.48	0.17
CZE	Czechia	0.53	0.55	0.19	0.27	0.15
ISL	Iceland	0.26	0.22	0.80	0.35	0.14
TUR	Turkey	0.42	0.34	0.56	0.15	0.11
SVK	Slovakia	0.38	0.11	0.40	0.37	0.10
BGR	Bulgaria	0.37	0.25	0.09	0.41	0.09
HRV	Croatia	0.23	0.41	0.19	0.37	0.08
ROU	Romania	0.31	0.12	0.13	0.46	0.08
GRC	Greece	0.19	0.37	0.18	0.34	0.07
HUN	Hungary	0.20	0.29	0.26	0.14	0.03
